# Selenocompounds as Novel Antibacterial Agents and Bacterial Efflux Pump Inhibitors

**DOI:** 10.3390/molecules24081487

**Published:** 2019-04-16

**Authors:** Tímea Mosolygó, Annamária Kincses, Andrea Csonka, Ádám Szabó Tönki, Karolina Witek, Carmen Sanmartín, Małgorzata Anna Marć, Jadwiga Handzlik, Katarzyna Kieć-Kononowicz, Enrique Domínguez-Álvarez, Gabriella Spengler

**Affiliations:** 1 Department of Medical Microbiology and Immunobiology, Faculty of Medicine, University of Szeged, Dóm tér 10, 6720 Szeged, Hungary; mosolygo.timea@med.u-szeged.hu (T.M.); kincses.annamaria@med.u-szeged.hu (A.K.); csonka.andrea83@gmail.com (A.C.); szabotadam@gmail.com (Á.S.T.); 2 Department of Obstetrics and Gynecology, Faculty of Medicine, University of Szeged, Semmelweis utca 1, 6725 Szeged, Hungary; 3 Department of Technology and Biotechnology of Drugs, Jagiellonian University Medical College, Medyczna 9, 30-688 Kraków, Poland; karolina.witek@uj.edu.pl (K.W.); marcmalgorzata@gmail.com (M.A.M.); j.handzlik@uj.edu.pl (J.H.); mfkonono@cyf-kr.edu.pl (K.K.-K.); 4 Department of Pharmaceutical Technology and Chemistry, School of Pharmacy and Nutrition, University of Navarra, Irunlarrea 1, 31008 Pamplona, Spain; sanmartin@unav.es; 5 Instituto de Investigación Sanitaria de Navarra (IdiSNA), Irunlarrea 3, 31008 Pamplona, Spain; 6 Interdisciplinary Excellence Centre, Department of Inorganic and Analytical Chemistry, University of Szeged, Dóm tér 7, 6720 Szeged, Hungary; 7 Instituto de Química Orgánica General (IQOG-CSIC), Consejo Superior de Investigaciones Científicas, Juan de la Cierva 3, 28006 Madrid, Spain

**Keywords:** selenocompounds, selenoesters, AcrAB-TolC efflux pump, *Chlamydia trachomatis* D, *Escherichia coli* K-12 AG100, *Staphylococcus aureus*

## Abstract

Bacterial multidrug resistance is becoming a growing problem for public health, due to the development and spreading of bacterial strains resistant to antimicrobials. In this study, the antibacterial and multidrug resistance reversing activity of a series of seleno-carbonyl compounds has been evaluated. The effects of eleven selenocompounds on bacterial growth were evaluated in *Staphylococcus aureus*, methicillin resistant *S. aureus* (MRSA), *Enterococcus faecalis, Escherichia coli*, and *Chlamydia trachomatis* D. The combination effect of compounds with antibiotics was examined by the minimum inhibitory concentration reduction assay. Their efflux pump (EP) inhibitory properties were assessed using real-time fluorimetry. Relative expressions of EP and quorum-sensing genes were studied by quantitative PCR. Results showed that a methylketone selenoester had remarkable antibacterial activity against Gram-positive bacteria and potentiated the activity of oxacillin in MRSA. Most of the selenocompounds showed significant anti-chlamydial effects. The selenoanhydride and the diselenodiester were active inhibitors of the AcrAB-TolC system. Based on these results it can be concluded that this group of selenocompounds can be attractive potential antibacterials and EP inhibitors. The discovery of new derivatives with a significant antibacterial activity as novel selenocompounds, is of high impact in the fight against resistant pathogens.

## 1. Introduction

Multidrug resistance is becoming a serious problem in the treatment of resistant bacterial infections. The discovery of novel antibacterial or multidrug resistance reversing agents is extremely urgent as soon we may lack effective drugs to treat bacterial infections caused by the arising superbugs resistant to the majority of the clinically available antibiotics [1]. Selenium (Se)-containing molecules could be possible alternatives in the development of a new approach to combat infections caused by multidrug resistant (MDR) pathogens. Se is an important element in biological molecules in archea, bacteria, and eukaryotes [2]. In humans, Se is an essential trace element and also has chemopreventive effects [3].

In this context, a few studies have reported that certain selenocompounds have shown an interesting antibacterial activity. First, a series of selenides-bearing benzenesulfonamide moieties has been found to strongly inhibit the carbonic anhydrases VchCAα and VchCAβ of *Vibrio cholerae*, thus, exerting an inhibition on the growth and pathogenicity of this bacterium [4]. In addition, a degraded selenide polysaccharide, extracted from *Enteromorpha prolifera*, has been found to show antibacterial activity against *Escherichia coli* [5]. Additionally, a series of fused selenazolinium salts have been shown to have a potent activity against **ESKAPE** pathogens, which are: vancomycin-resistant **E**nterococci, methicillin-resistant ***S****taphylococcus aureus* (MRSA), ***K****lebsiella pneumoniae, **A**cinetobacter baumannii, **P**seudomonas aeruginosa*, and carbapenem-resistant ***E**nterobacteriaceae*. The majority of these compounds have minimum inhibitory concentration (MIC) values below 1 µg/mL, in resistant bacterial strains of MRSA, *K. pneumoniae*, *A. baumanii*, and *P. aeruginosa* [6]. This last work highlights the potential applications of selenocompounds, in the treatment of infections caused by the MDR bacterial strains. Our previous studies have demonstrated that the selenoanhydride **1** and selected selenoesters **2**–**11** (Table 1) have shown potent anticancer activity against ATP-Binding cassette sub-family B member 1 (ABCB1)-overexpressing MDR mouse T-lymphoma cells and MDR colon adenocarcinoma cells [7,8]. The ABC family of protein transporters also plays an important role in bacterial multidrug resistance [9]. Several members of the ABC family, e.g., MsrA in staphylococci [10,11] or Msr(D) in *Streptococcus pneumoniae* [12], significantly contribute to the efflux of antibiotics, and are considered as attractive protein targets in experimental chemotherapy. A major factor of bacterial and cancer drug resistance is assigned to the MDR efflux transporter proteins, expelling toxic compounds and drugs out of the cells. Based on the energy source of these pumps, the primary transporter derives their energy from the hydrolysis of ATP (ABC-transporters) and secondary transporters use proton or ion gradients to drive the extrusion of toxic compounds. Using selenocompounds, such as chemosensitizers, these compounds have been shown to inhibit the ABCB1 in cancer cells [7,8], and based on these results, our aim was to investigate the efflux pump inhibitory properties of these selenocompounds on the representative bacterial efflux system AcrAB-TolC. RND (Resistance–Nodulation–Division) family transporters are widespread, especially among Gram-negative bacteria, and catalyse the efflux of antibiotics and biocides. This tripartite efflux system consists of an outer membrane channel and periplasmic adaptor proteins, and the inner membrane transporter AcrB [13]. The MarR transcription factor regulates resistance to diverse antibiotics, organic solvents and oxidative stress agents by controlling the expression of efflux pumps (including AcrAB-TolC) through the repression of the operon that encodes the transcriptional activator MarA. The antibiotic resistance arises when the MarR protein is inactivated or the expression of *marR* genes is inhibited [14]. Although the expression of AcrAB-TolC efflux pump is regulated at several levels, the MarR the AcrR also regulates it negatively, meanwhile, the MarA, SoxS, and Rob are activators of this efflux pump [15]. In addition, the quorum sensing (QS) regulators, such as SdiA could also affect the expression of AcrAB-TolC efflux pump in *E. coli*, since AcrAB-TolC has been proposed to pump out QS signals [16].

Furthermore, coating surfaces with Se could reduce the bacterial attachment to prosthetic devices [17], whereas sodium selenite exhibited ulcer healing and antibacterial activity against *Helicobacter pylori* [18]. Various studies have highlighted the antimicrobial properties of elemental Se, in the form of nanoparticles (SeNP) against *S. aureus* [19,20,21,22], *Staphylococcus epidermidis*, *K. pneumoniae*, *Bacillus subtilis* [23], *P. aeruginosa*, *E. coli*, and *A. baumannii* [24]. Additionally, biogenic SeNPs, synthesized by different non-pathogenic bacterial strains and stabilized with bacterial proteins, have shown activity against pathogenic bacteria [25,26].

In addition, there is an emerging evidence that *Chlamydia trachomatis* is developing resistance to antibiotics, as certain clinical isolates have shown single- or multidrug resistance [27,28]. Consequently, the development of new antibacterials and multidrug resistance reversing compounds is required to overcome this emerging problem. Although there are numerous studies that have investigated the antibacterial activity of Se-containing (in)organic compounds and SeNPs, according to our knowledge, no report has been described regarding anti-chlamydial activity of selenocompounds. Furthermore, the selenocompounds found as anticancer agents and cancer efflux pump inhibitors have not yet been tested on any bacterial strains.

Herein, we report the antibacterial effects of selenocompounds **1**–**11** on Gram-negative and Gram-positive bacteria, such as *E. coli*, *C. trachomatis* D, *Enterococcus faecalis*, and *S. aureus* (including methicillin resistant strain, MRSA).

## 2. Results

### 2.1. Antibacterial Activity: Determination of the MIC

The ketone-containing selenoesters **9**–**11** showed a potent antibacterial activity against the Gram-positive *S. aureus* ATCC 25923 and MRSA HEMSA 5. The methylketone selenoester **9** was the most active agent with noteworthy MIC values in the low micromolar range (3.12 and 3.91 µM). The *tert*-butylketone selenoesters **10** and **11** showed lower antibacterial activity than methylketone selenoester **9**, but was still significant (25 and 50 µM). The selenoanhydride **1** and the remaining selenoesters **2**–**8** evaluated were inactive as their MIC were equal or above 100 µM. The selenoester **9** showed also significant antibacterial activity towards *E. faecalis*, but this Gram-positive strain was less sensitive to **9** than *S. aureus* and MRSA (MIC = 12.5 µM), and also was not sensitive to the rest of the selenocompounds tested (MIC > 100 µM), (Table 2).

In contrast, none of the eleven Se derivatives demonstrated antibacterial effects against the two Gram-negative strains evaluated in this study, which are the AcrAB-TolC-expressing *E. coli* AG100 and the AcrAB-TolC-deleted mutant *E. coli* AG100A. In these two strains, all compounds showed MIC values above 100 μM (data not shown).

### 2.2. Enhancement of the Activity of Antibiotics

In order to determine if selenocompounds **1**–**11** enhance the activity of antibiotics, they were tested in combination with antibiotics commonly used in clinical therapy, which are substrates of the AcrB pump—tetracycline [29] and ciprofloxacin [30]. The combined effects of selenocompounds and these antibiotics were tested on the AcrAB-TolC expressing Gram-negative *E. coli* AG100 strain. In addition, the chemosensitizing effects of selenocompounds on the Gram-positive MRSA HEMSA 5 strain were studied in combination with oxacillin. These antibiotics have been selected among the ones that are more widely used in clinical practice, in an attempt to cover different mechanisms of action, to see which ones are more affected by the selenocompounds. Gram-negative efflux pumps of the RND superfamily in Gram-negative bacteria are crucial to the cellular defence mechanisms, but the overexpression of these pumps can lead to multidrug resistance, which is an alarming problem for health care. The AcrAB-TolC system containing the RND type pump AcrB has been studied extensively, due to its importance in bacterial resistance. As an in vitro model system we used the AcrAB-TolC overexpressing *E. coli* AG100 strain and its pump-deleted mutant strain *E. coli* AG100A, in order to find effective efflux pump inhibitor (EPI) compounds. EPIs as chemosenzitizers could reverse the resistant phenotype, and in combination with antibiotics, they could enhance the activity of these conventional antibiotics. Moreover, the Gram-positive methicillin resistant *Staphylococcus aureus* (MRSA) is a major concern in healthcare facilities, for this reason our aim was to test the selenocompounds on reference ATCC and resistant MRSA strains. The enhancement of the activity of oxacillin was studied as the ability of compounds **1**–**11**, to reduce MIC of oxacillin against MRSA, whereas the enhancement of tetracycline or ciprofloxacin was studied analogously for AcrAB-TolC-expressing *E. coli* strain (Table 3).

In the absence of the selenocompounds, oxacillin showed MIC value of 374 µM (150 μg/mL) against MRSA, since this strain was highly resistant to this β-lactam antibiotic. The methylketone selenoester **9**, at a low concentration of 1.95 μM (0.537 μg/mL), exerted a noteworthy 64-fold reduction of the MIC value of oxacillin to 5.84 µM (2.34 μg/mL). Hence, this compound can be useful as a potent agent to reverse the resistance of MRSA to oxacillin. Interestingly, the rest of the compounds **1**–**8**, **10**, and **11** were not active, even at the concentration of 62.5 μM (MIC reduction of oxacillin ≤ 2).

However, none of the tested selenocompounds were able to improve the efficacy of antibiotics against the AcrAB-TolC-overexpressing strain of *E. coli* with the above-mentioned excellent 64-fold factor. The highest reductions observed in *E. coli* were a 2-fold reduction of the MIC values of tetracycline (compound **9**) and of ciprofloxacin (compounds **9, 10**).

### 2.3. Anti-Chlamydial Activity

**Figure 1:**
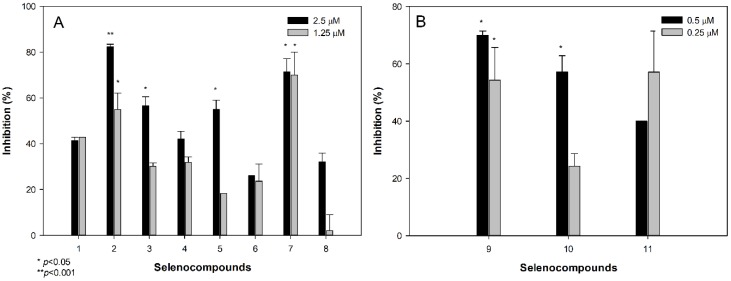
Anti-chlamydial effect of selenocompounds at 1.25 and 2.5 μM (**A**), and at 0.25 and 0.5 μM (**B**).

Before the assessment of the anti-chlamydial activity of the selenocompounds, a cytotoxicity assay was performed on HeLa cells to determine the ranges of concentrations at which the selenocompounds can be evaluated without showing direct toxic effects to HeLa cells. Selenocompounds **2**, **3**, **5**, **7**, and **9**–**11**, significantly inhibited the formation of chlamydial inclusions at selected concentrations (Figure 1).

Compounds **2** and **7** at 2.5 μM, showed 82% and 71% inhibition, compared to the control, respectively. In addition, **2** and **7** were effective at 1.25 μM, whereas **9** and **10** inhibited the formation of inclusions at low submicromolar concentrations of 0.5 μM. The most potent anti-chlamydial selenocompounds were **9** and **11**, as they inhibited more than 50% of the growth of *C. trachomatis* D, at a concentration of 0.25 μM (0.0689 and 0.0858 μg/mL, respectively). 

### 2.4. Real-Time Accumulation Assay

Since ethidium bromide (EB) is a substrate of the AcrB efflux pump, the intracellular accumulation of EB provides information about the inhibition of the AcrAB-TolC system, in the presence of selenocompounds, in a time-dependent manner. The assay records the real-time accumulation of EB, using a real-time thermocycler, by monitoring the fluorescence of EB inside the cells [31]. The activities of compounds **1**–**11** in the real-time EB accumulation assay, were given in terms of the relative fluorescence index (RFI) of the real-time accumulation curves (Table 4). In case of the real-time EB accumulation, the amount of EB accumulated by cells was higher if the difference between RF_treated_ and RF_untreated_ was greater, therefore, the degree of inhibition of the efflux pump system by the compound became greater. Compounds **9** and **10** possessed EPI activity and decreased the MIC of ciprofloxacin on *E. coli* AG100. However, the selenoanhydride **1** and the selenoester **4**, compared with the positive control promethazine (PMZ, RFI: 0.15), strongly inhibited the efflux of AcrAB-TolC in *E. coli* AG100; they had no effect in combination with the antibiotics, suggesting that other cellular mechanisms might also be involved in the mode of action, such as interaction with cell wall components, formation of reactive oxygen species (ROS), or membrane destabilizing effects. Without investigating the possible metabolites of the selenocompounds, no further conclusions can be drawn, for this reason we are planning to study the metabolites of these compounds in future works. Derivatives **7** and **9**–**11** caused moderate inhibitory action, whereas **2**, **3**, **5**, **6** and **8** showed weak or no activity on the intracellular EB accumulation in *E. coli* AG100. Among derivatives **7** and **9**–**11**, compound **7**, which contained a methyl oxoester in the alkyl moiety bound to Se, was the most active agent (RFI = 0.13).

Nevertheless, no efflux pump inhibitory action of selenocompounds (**1**–**11**) was found in the *E. coli* AG100A strain.

### 2.5. Gene Expression Analysis by Quantitative PCR

**Figure 2:**
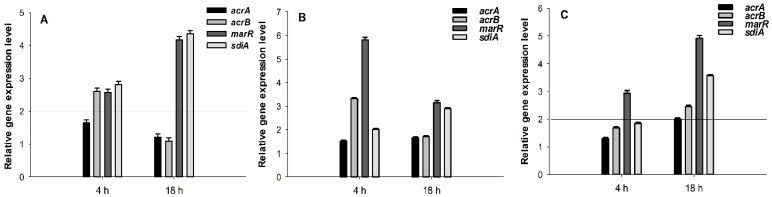
Relative gene expression levels of the genes of the *acrA*, *acrB*, *marR*, and *sdiA* in the presence of compounds **1** (**A**), **4** (**B**), and **7** (**C**), after 4 and 18 h exposure. The line denotes a threshold value, which was set at a two-fold increase in transcripts.

For the effect of the selenocompounds on the relative expression of the efflux pump, antibiotic resistance and QS genes in *E. coli* AG100—the most effective compounds in the EB real-time accumulation assay—were examined (compounds **1**, **4**, **7**; Figure 2). In this assay, the genes of the multidrug efflux pump (AcrAB), the component of the *E. coli mar* locus (multiple antibiotic resistance), and the gene of SdiA were investigated. The changes in gene expression from reverse transcription quantitative PCR experiments were normalized to the expression of *gapdh* (internal control), in the same sample, and compared to the expression of the examined genes obtained from the untreated, control samples.

As shown in Figure 2A, compound **1** at 50 μM concentration significantly up-regulated the *acrB*, *marR*, and *sdiA* genes, after 4 h of exposure. However, after 18 h, the expression of the *acrB* gene returned to the basal levels and the expression of the *marR* and *sdiA* genes, significantly increased. Compound **4** up-regulated the expression levels of *acrB*, *marR*, and *sdiA*, after 4 h, although after 18 h, the expression levels of the *acrB* and *marR* genes decreased. The QS gene *sdiA* was significantly up-regulated after 18 h (Figure 2B).

Compound **7** also significantly up-regulated *marR*, after exposures of 4 h and of 18 h. After 18 h, the expression level of the RND transporters subunit genes (*acrA*, *acrB*) was significantly increased (Figure 2C). 

## 3. Discussion

Results of these studies indicate that selenoesters and selenoanhydrides, previously found as active anticancer or ABCB1 efflux pump inhibitors in cancer cells [7,8,32,33,34,35], also displayed a promising antimicrobial potential against the MDR bacterial strains.

### 3.1. Antibacterial Activity

The evaluation of the compounds proved that the ketone-containing selenoesters **9**–**11** showed an antibacterial activity against the Gram-positive reference *S. aureus* strain, whereas, the methylketone selenoester **9** was also active against the MRSA HEMSA 5 and *E. faecalis*. However, none of the compounds were active against the Gram-negative *E. coli* ag100. The background of the different antibacterial activity of **9** against the tested Gram-positives and Gram-negatives was unknown; further experiments are required to clarify whether the mechanism of action of the methylketone selenoester could be related with any kind of interaction between this compound and the bacterial cell wall that is typical for gram-positive bacteria. In contrast, the remaining alkyl groups (–CH_3_) or alkyl-functionalized moieties (–CH_2_CONH_2_, –CH_2_COOCH_3_ and –CH_2_COOPh, Table 1) bound to the Se atom rendered selenoesters that were ineffective against the tested strains. Interestingly, the compounds **9**–**11** were also the most potent anticancer agents in previous works [7,8,33], and they also showed a good selectivity towards cancer cells, with respect to non-tumour cell lines, as they showed selectivity indexes ranging from 8.4 to 14.4 [8].

In previous works, it was hypothesized that the possible mechanism of action of these compounds could be the hydrolysis of the compound and the subsequent liberation of the ionic species of Se, which could be responsible for the activity of the compounds [33]. In this case, this phenomenon enables us to hypothesize that the CH_3_COCH_2_SeH, or its anionic form, are the chemical forms of Se that could be behind the observed activities. The lack of activity of the non-ketone selenoesters, directs a special attention to this –SeCH_2_COCH_3_ ketone-containing moiety. 

### 3.2. Enhancement of the Activity of Antibiotics

The activity of **9** on the MDR clinical isolate (MRSA) was very promising, because compound **9** reduced the MIC of oxacillin in 64-fold (from 374 µM to 5.84 µM). These results supported the potential applications of the methylketone selenoesters, such as antimicrobials, and the multidrug resistance reversing agents. These results were in accordance with the activity shown by these compounds as enhancers of the anticancer activity of chemotherapy drugs [35], suggesting that these selenium derivatives have the ability to effectively interact with the resistance mechanisms developed by the resistant bacterial strains and by the resistant cancer cells. This work intends to carry out a screening of the potential applications of the selenocompounds, and in future works we will attempt to ascertain the possible mechanisms of actions of the activities described herein, as this observed potential enhancement of the activity of oxacillin exerted by compound **9** in an MRSA clinical isolate.

### 3.3. Anti-Chlamydial Activity

Previous studies have reported that selected selenocompounds, such as certain selenocyanates, selenoureas, and diselenides, showed antiproliferative activities against the intracellular forms of *Leishmania spp.* [36,37]. Taking those results into account, this study provided a new line of evidence for the action of selenoanhydride/selenoesters on an obligate intracellular chlamydial strain. In particular, different selenoesters, such as **2**, **3**, **5**, **7**, and **9**–**11**, have exerted a noteworthy activity against *C. trachomatis* D. Furthermore, the activities of the methyl (**9**) and the *tert*-butyl (**11**) derivatives were very promising, as they inhibited the formation of more than 50% of the chlamydial inclusions, at a very low concentration (0.25 μM). However, their mode of action has not been ascertained in this study. 

Regarding the observed structure activity relationships of the anti-chlamydial assays, the ketone selenoesters **9**–**11** showed noteworthy activity at lower concentrations (0.25 μM, 0.5 μM), compared to the rest of the series (1.25 μM, 2.5 μM). Among the remaining selenoesters, the symmetric dimethyl selenodiester, which contains a thiophene ring **2**, and the methyl oxoester derivative **7** showed a better activity, and the activities of the symmetric dimethyl selenodiesters **3** and **5** were also remarkable. These fact highlights the importance of the symmetry for the activity against intracellular pathogens [36].

### 3.4. Interaction of the Compounds with Bacterial Efflux Pumps

The resistance to the current antibacterial drugs is one of the major therapeutic challenges in the treatment of bacterial infections, and knowing the potential of these derivatives as multidrug resistance reversing agents (proved both by the capacity to enhance the activity of antibiotics described above and by the enhancement of anticancer drugs reported in previous works), we have studied here the procedure through which selenocompounds interact with the bacterial AcrAB-TolC system in the *E. coli* AG100 strain. 

The results obtained revealed that the cyclic selenoanhydride **1** significantly inhibited this bacterial AcrAB-TolC efflux pump in the *E. coli* AG100 strain. Similarly, EP inhibiting activity has been found for compounds **4** and **7**. The second most potent inhibitor was the symmetrical benzene derivative 1,3-disubstituted with methylselenoester moieties (**4**). Interestingly, its 1,4-disubstituted analogue (**5**) showed an EP-inhibitory activity, 4.5-fold lower, suggesting the importance of the substituents’ topology for the expected biological effect. Taking into account the distinct difference in electron density properties between *m-* and *p-*substituted phenyl rings, this factor seems to have affected the mechanisms of EP inhibition.

The well-characterized RND-type transporter, AcrB is associated with TolC and AcrA and is the major efflux pump of *E. coli* [38]. These efflux pumps recognize and extrude a large variety of antibiotics from the cytoplasm. The energy required for the operation of the efflux pump is provided by the proton motive force, created by the proton gradient resulting from electron transport [39]. This fact suggests that those selenocompounds, which possessed EP inhibitor activity, might interfere with the proton motive force. Surprisingly, compounds **1** and **4**, which inhibited the AcrAB-TolC system, influenced the expression of the gene *acrB*, which is a constituent of the AcrAB-TolC system. In addition, the compounds increased the expression of the QS gene *sdiA*, after 18 h of exposure, which suggests their roles in QS, although their QS inhibitory activities were not investigated in this study.

## 4. Materials and Methods 

### 4.1. Chemistry

Eleven pure selenocompounds obtained as described earlier [33], were examined (**1**–**11**, Table 1). All compounds were stable in air and their purity was assessed by elemental analysis and ^1^H and ^13^C NMR, as reported in a previous work [35]. Before their use in biological assays, they were dissolved in dimethyl sulfoxide (DMSO; Merck KGaA, Darmstadt, Germany), to obtain stock solutions. Working solutions were prepared by dilutions in the culture medium.

### 4.2. Bacterial Strains

Wild-type *E. coli* K-12 AG100 strain [argE3 thi-1 rpsL xyl mtl Δ(gal-uvrB) supE44] and its AcrAB-TolC-deleted mutant strain *E. coli* AG100A (a kind gift from Hiroshi Nikaido, Department of Molecular and Cell Biology and Chemistry, University of California, Berkeley, USA) were used for the evaluation of the EPI activity of the tested selenocompounds. 

*S. aureus* ATCC 25923 and *E. faecalis* ATCC 29212 strains were used to determine the MIC. A methicillin-resistant *S. aureus* strain (MRSA HEMSA 5, a clinical isolate) was used in the combination assay, with oxacillin, to determine the capacity of compounds to enhance the antibacterial effect of this antibiotic. *C. trachomatis* reference strain (serovar D, UW-3/Cx, ATCC, VR-885D) was used in the anti-chlamydial assay.

### 4.3. Propagation of C. trachomatis D

*C. trachomatis* D was propagated on the HeLa 229 cells (ATCC, CCL-2.1), as described earlier [40]. The titre of the infectious elementary bodies was determined by an indirect immunofluorescence assay. Serial dilutions of the elementary bodies’ preparation were inoculated onto the HeLa monolayers and, after a 48-h culture, the cells were fixed with acetone, and stained with monoclonal anti-*Chlamydia* LPS antibody (AbD Serotec, Oxford, UK) and FITC-labelled anti-mouse IgG (Merck KGaA, Darmstadt, Germany). The inclusions of *C. trachomatis* D were enumerated under a UV microscope.

### 4.4. Determination of MIC

The effects exerted by different concentrations of the compounds on the bacterial growth in *S. aureus*, *E. faecalis*, and *E. coli* AG100 were tested in 96-well plates. The MICs of selenocompounds were determined, according to the Clinical and Laboratory Standard Institute (CLSI) guidelines [41]. The DMSO exerted no antibacterial effect. Alternatively, the MIC of the oxacillin in MRSA HEMSA 5 was determined by the broth microdilution method, in a cation-adjusted Mueller–Hinton Broth (MHB II), according to the recommendations of the CLSI. Results were recorded after a 20- or 24-hour incubation at 37 °C.

### 4.5. Enhancement of the Activity of Antibiotics

The chemosensitizing effect of the tested selenocompounds was evaluated through the determination of the MIC values of the antibiotics, in the presence of sub-inhibitory concentrations of the compounds (MIC/2 or MIC/4), in both Gram-negative (*E. coli* AG100) and Gram-positive (MRSA) strains. The MICs were evaluated in the *E. coli* strain, by a two-fold broth microdilution method in the 96-well plates, using serial dilutions of tetracycline (from 8.4 to 0.16 μM) and ciprofloxacin (from 1.4 to 2.7 × 10^−3^ μM). The first four rows contained two-fold dilutions of antibiotics, and the combinations of the antibiotics and tested compounds were added into the last four rows. 10^−4^ dilution of an overnight bacterial culture in 50 μL of MHB was then added to each well, with the exception of the medium control wells. The plates were then incubated at 37 °C for 18 h. MIC values of the antibiotics and their combination with the tested compounds were determined by naked eyes. In the assay with oxacillin in the MRSA HEMSA 5 bacterial strain, a microdilution method in MHB II was used.

### 4.6. Anti-Chlamydial Assay

Elementary bodies of *C. trachomatis* D (4 × 10^3^ IFU/mL) were incubated with compounds at selected concentrations (0.25, 0.5, 1.25, 2.5 μM) in sucrose-phosphate-glutamic acid buffer (SPG) for 2 h at 37 °C. As a control, *C. trachomatis* D was also incubated alone in the SPG. To quantify the anti-chlamydial effects of the compounds, HeLa cells were seeded in 24-well plates with 13-mm cover glasses. The confluent cells were infected with compound-treated *C. trachomatis* D or with the non-treated controls. After 48 h, the cells were fixed with acetone at −20 °C for 10 min. The titre of the infectious elementary bodies was determined by the indirect immunofluorescence assay, as described earlier [42]. 

### 4.7. Real-Time Accumulation Assay 

The effect of the studied selenocompounds on the real-time accumulation of ethidium bromide (EB) was assessed by an automated EB method [43], using a LightCycler real-time thermocycler (LightCycler 1.5; Roche). The compounds were added individually at different concentrations at MIC/2 to the EB solution in PBS. The final concentration of EB was 1 and 0.25 µg/mL for *E. coli* AG100 and AG100A, respectively. The method for the calculation of the relative fluorescence index (RFI) of the last time point (minute 30) was described earlier by Kincses et al. [44]. Promethazine (PMZ; EGIS) was applied as a positive control.

### 4.8. Expression Analyses of Genes by Quantitative PCR

Total RNA was isolated from *E. coli* AG100 (OD of 0.6 at 600 nm) using the NucleoSpin RNA kit (Macherey Nagel) according to the manufacturer’s instructions. The concentration of the extracted RNA templates was assessed by spectrophotometry at 260 nm. 

The expression of the *acrA*, the *acrB*, the multiple antibiotic resistance protein R (*marR*), and the quorum-sensing transcriptional activator (*sdiA*) genes was studied by reverse transcription quantitative PCR (RT-qPCR), as described earlier [44]. The real-time one-step PCR was performed in a CFX96 Touch real-time PCR detection system (Bio-Rad), strictly adhering to the manufacturer recommendations of the SensiFAST™ SYBR No-ROX One-Step Kit (Bioline GmbH, Luckenwalde, Germany). The forward and reverse primers used in the experiment are shown in Table 5 [44,45]. The cycle threshold (Ct) values were determined with the Bio-Rad CFX Manager Software version 3.1. Relative quantification analysis was carried out using the Livak method [46]. The expression of *gapdh* was used as the internal control and the untreated *E. coli* AG 100 served as the external control. We have defined a threshold value—increases greater than 2-fold in the amount of transcripts relative to the control samples were considered significant.

## 5. Conclusions

Herein, we have reported the evaluation of the antibacterial and multidrug resistance reversing activity of 11 novel selenocompounds. The most active compound in the antibacterial assay, the methylketone selenoester **9**, showed potential antibacterial activity against the different strains of *S. aureus, E. faecalis*, and *C. trachomatis* D, even at very low concentrations (0.25 μM for *C. trachomatis* D). This selenocompound also enhanced the efficacy of antibiotics, namely it multiplied by 64-fold the antibacterial action of oxacillin, against the MDR clinical isolate of *S. aureus*. Alternatively, three compounds (the selenoanhydride **1** and the selenoesters **4** and **7**) inhibited the tripartite multidrug resistance efflux pump AcrAB-TolC in *E. coli*, and affected the expression of the different genes related to these resistance processes. 

Based on these results, it can be concluded that this group of selenocompounds can be attractive potential EP inhibitors and antibacterial lead scaffolds, for further development of new chemical tools, to overcome bacterial multidrug resistance.

## Figures and Tables

**Table 1:** Selenocompounds evaluated as antibacterial and as multidrug resistance reversing agents—selenoanhydride (**1**) and selenoesters (**2**–**11**).

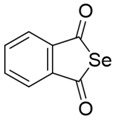			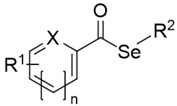		
**I**. Cyclic selenoanhydride (**1**)			**II**. Selenoesters (**2**–**11**)		
**Compound**	**Group**	**R^1^**	**X**	**n**	**R^2^**
**1**	**I**	-	-	-	-
**2**	**II**	5-COSeCH_3_	S	0	-CH_3_
**3**	**II**	6-COSeCH_3_	N	1	-CH_3_
**4**	**II**	3-COSeCH_3_	C	1	-CH_3_
**5**	**II**	4-COSeCH_3_	C	1	-CH_3_
**6**	**II**	-H	C	1	-CH_2_CONH_2_
**7**	**II**	4−Cl	C	1	-CH_2_COOCH_3_
**8**	**II**	-H	C	1	-CH_2_COOPh
**9**	**II**	4−Cl	C	1	-CH_2_COCH_3_
**10**	**II**	4−Cl	C	1	-CH_2_COC(CH_3_)_3_
**11**	**II**	3,5-diOCH_3_	C	1	-CH_2_COC(CH_3_)_3_

**Table 2:** Minimum inhibitory concentration (MICs) of the selenocompounds on the Gram-positive bacteria. In bold—MIC values <10 μM.

Compounds	MIC (μM)		
	*Staphylococcus aureus* ATCC 25923	*Staphylococcus aureus* HEMSA 5	*Enterococcus Faecalis* ATCC 29212
**1**	>100	>125	>100
**2**	100	>125	>100
**3**	100	>125	>100
**4**	100	>125	>100
**5**	>100	>125	>100
**6**	>100	>125	>100
**7**	100	125	>100
**8**	100	>125	>100
**9**	**3.12**	**3.91**	12.5
**10**	25	>125	>100
**11**	50	>125	>100

**Table 3:** Numerical value of the reduction of the MICs of selected antibiotics in methicillin resistant *S. aureus* (MRSA) or in *E. coli* AG100 exerted by selenocompounds when administered in combination with antibiotics.

Cpd^1^	MRSA HEMSA 5		*Escherichia coli* AG100		
	Concentration of Compound [µM]^2^	Reduction of Oxacillin MIC	Concentration of Compound [µM]	Reduction of Tetracycline mic	Reduction of Ciprofloxacin mic
**1**	62.5	no effect	50	no effect	no effect
**2**	ND	ND ^3^	50	no effect	no effect
**3**	62.5	2-fold	50	no effect	no effect
**4**	62.5	no effect	50	no effect	no effect
**5**	62.5	no effect	50	no effect	no effect
**6**	62.5	no effect	50	no effect	no effect
**7**	62.5	≥ 2-fold	50	no effect	no effect
**8**	62.5	2-fold	50	no effect	no effect
**9**	1.95	64-fold	25	2-fold	2-fold
**10**	62.5	no effect	50	no effect	2-fold
**11**	62.5	no effect	50	no effect	no effect

^1^ Cpd: Compound. ^2^ Starting concentration of tetracycline: 8.4 μM; ciprofloxacin: 1.4 μM; and oxacillin: 747 μM. ^3^ ND: Not determined.

**Table 4:** Relative fluorescence index (RFI) for the effect of selenocompounds and positive control promethazine (PMZ) on the AcrAB-TolC-expressing *Escherichia coli* AG100 strain.

Compound	RFI^a^	Compound	RFI^a^	Compound	RFI^a^
	*Escherichia coli* AG100		*Escherichia coli* AG100		*Escherichia coli* AG100
**1**	0.28	**5**	0.04	**9**	0.11
**2**	0.03	**6**	0.06	**10**	0.12
**3**	0.04	**7**	0.13	**11**	0.11
**4**	0.18	**8**	0.08	**PMZ**	0.15

**Table 5:** Primers used in the RT-qPCR.

Gene	Full Name	Primer Sequence (5’–3’)	Amplicon size (bp)	Ref.
***acrA***	Acridine resistance protein A	CTTAGCCCTAACAGGATGTG TTGAAATTACGCTTCAGGAT	189	[45]
***acrB***	Acridine resistance protein B	CGTACACAGAAAGTGCTCAA CGCTTCAACTTTGTTTTCTT	183	[45]
***marR***	Multiple antibiotic resistance protein R	AGCGATCTGTTCAATGAAAT TTCAGTTCAACCGGAGTAAT	170	[45]
***sdiA***	Quorum-sensing transcriptional activator	CTGATGGCTCTGATGCGTTTA TCTGGTGGAAATTGACCGTATT	163	[44]
***gapdh***	Glyceraldehyde-3-phospate dehydrogenase	ACTTACGAGCAGATCAAAGC AGTTTCACGAAGTTGTCGTT	170	[45]
